# Case Report: Haemopoietic stem cell transplantation in refractory paediatric RAS-associated lymphoproliferative disorder

**DOI:** 10.3389/fimmu.2026.1850700

**Published:** 2026-06-09

**Authors:** Srividhya Senthil, Michail G. Matalliotakis, Stephen Hughes, Peter D. Arkwright, Mohan Shenoy, Robert Wynn

**Affiliations:** 1 Department of Paediatric Bone Marrow Transplant, Royal Manchester Children’s Hospital, Manchester, United Kingdom; 2 Department of Paediatric Allergy and Immunology, Royal Manchester Children’s Hospital, Manchester, United Kingdom; 3 Lydia Becker Institute of Immunology and Inflammation, University of Manchester, Manchester, United Kingdom; 4 Department of Paediatric Nephrology, Royal Manchester Children’s Hospital, Manchester, United Kingdom

**Keywords:** HSCT, MEK inhibition, MEK inhibitor, paediatric, RALD, RAS-associated autoimmune leukoproliferative disorder (RALD), stem cell transplant (SCT)

## Abstract

RAS-associated lymphoproliferative disorder (RALD), caused by acquired gain-of-function mutations in Kirsten rat sarcoma KRAS or Neuroblastoma rat sarcoma NRAS, is an orphan disease with very few cases reported worldwide. The most common presenting feature is autoimmune cytopenia, which is generally documented to be responsive to immunosuppression. We describe two cases of RALD in which associated autoimmunity proved refractory to multiagent immunosuppression. We also share our experience of haematopoietic stem cell transplantation (HSCT) in the two patients with recalcitrant autoimmunity, adding valuable insight into the distinctive complications and transplant course in this rare disorder. Furthermore, we review the published literature on HSCT in RALD, which includes three other patients, primarily transplanted for malignant transformation of RALD. In addition, we report our experience with targeted therapy, specifically the MEK inhibitor, in this disease and reflect on its role within the treatment paradigm of RALD.

## Introduction

Rat sarcoma (RAT) RAS-associated lymphoproliferative disorder (RALD) (OMIM No. 614470) is caused by acquired gain-of-function mutations in either *Kirsten rat sarcoma* (*KRAS*) or *Neuroblastoma rat sarcoma* (*NRAS*), leading to defects in intrinsic apoptotic pathways within lymphoid and myeloid lineages. It is a chronic disorder, and its clinical presentation resembles autoimmune lymphoproliferative syndrome (ALPS), with autoimmunity, lymphadenopathy, and hepatosplenomegaly (1). Transformation to juvenile myelomonocytic leukaemia (JMML) and acute leukaemia has also been reported (2). The management of RALD has primarily relied on immunosuppression, and there are no published reports of haematopoietic stem cell transplantation (HSCT) for treatment-refractory autoimmunity in this condition. We report two challenging RALD cases with refractory autoimmunity treated with judicious HSCT. Both patients experienced graft rejection after their first transplant, complicated by likely disease-specific factors, and required a second HSCT. Here, we review the transplant experience and consider the role of HSCT in this rare disease.

## Case descriptions

### Patient 1

An 18-month-old man of Asian ethnicity presented with fever, pallor, and bruising and was found to have a direct antiglobulin test (DAT)-positive autoimmune haemolytic anaemia and immune thrombocytopenia. Total white cell count was elevated (36 × 10^9^/L), with a monocytosis of 4 × 10^9^/L. He had gross splenomegaly but no lymphadenopathy. Blood smear revealed a leucoerythroblastic blood picture with monocytosis and lymphocytosis, immature myeloid and erythroid precursors, spherocytes, and polychromasia. Haemoglobin F (HbF) was raised for age. Clinical suspicion for JMML was raised, and bone marrow assessment revealed a hypercellular marrow with no increase in blast population and a normal karyotype. Fluorescent *in situ* hybridisation (FISH) was negative for Breakpoint Cluster Region – Abelson murine leukaemia Breakpoint Cluster Region - Abelson murine leukaemia (BCR-ABL) translocation.

He had hypergammaglobulinaemia with reduced complement C3 and C4 levels. T and B lymphocyte subsets in peripheral blood showed that only 0.02% of CD3, TCR alpha/beta-positive T cells were double-negative (CD4/CD8). Additionally, high titres of antinuclear antibodies (ANA) and anti-double-stranded DNA (anti-dsDNA) antibodies were detected. **Table 1** lists the remainder of the initial investigations undertaken at presentation. These features raised suspicion of an underlying primary immune dysregulatory disorder (PIRD), prompting further investigations. Myeloid next-generation sequencing (NGS; included canonical mutations of JMML, i.e., PTPN11, NF1, CBL, KRAS, and NRAS) from peripheral blood revealed a heterozygous somatic *NRAS* gain-of-function variant (c.38G>A; p{Gly13Asp}) with a variant allele frequency (VAF) of 45%. The variant was absent in skin fibroblasts, thereby excluding a germline RAS mutation. No other abnormality was detected on myeloid NGS.

**Table 1:** Results of initial investigation in the reported patients.

Investigations (presentation)	Patient 1	Patient 2	Reference range
Haemoglobin	50	78	101–138 g/L
MCV	107	74	73–88 fL
Platelets	11	3	150–550 × 10^9^/L
White cell count	36.1	9.7	6.0–17.0 × 10^9^/L
Neutrophils	NA	1.80	1.00–8.50 × 10^9^/L
Lymphocytes	NA	4.18	1.80–10.50 × 10^9^/L
Monocytes	4.0	2.4	0.10–1.30 × 10^9^/L
Creatinine	28	17	15–31 µmol/L
CRP	4	32	0.3–5.0 mg/L
Ferritin	76	66	30–400 µg/L
ESR	34	NA	4–10 mm/first hour
C3	0.34	NA	0.62–1.6 g/L
C4	0.02	NA	0.14–0.39 g/L
Immunoglobulins	IgG 50.30	IgG 19.58	IgG 3.1–13.8 g/L
	IgA 2.83	IgA 0.93	IgA 0.3–1.3 g/L
	IgM 2.66	IgM 1.28	IgM 0.5–1.9 g/L
ANA	Positive^a^	Negative	–
Anti-dsDNA	> 300	1.0	0–9.9 IU/ml
Spleen	Enlarged (11.7 cm on abdominal USS)	Enlarged (10.2 cm on abdominal USS)	–
Liver	Enlarged (11.4 cm on abdominal USS)	Enlarged (on abdominal USS)	–
Lymphadenopathy	Present	Absent	–
Urine protein: creatinine	0	NA	–

^a^ ANA pattern for patient 1, centromere negative; *Crithidia* positive; Ss-A antibody negative; Ss-A52 antibody negative; Ss-A60 antibody negative; Ss-B antibody negative; Rnp 68 negative; anti-Sm negative; Smrnp antibody negative; ribosomal P positive > 8.0 IU/ml (reference range: 0–0.9); chromatin positive > 8.0 IU/ml (reference range: 0–0.9); Jo-1 negative; Scl-70 negative; CCP IgG antibody positive, 6.3 kIU/L (reference range: 0–2.9).

*NA*, not available.

The immune cytopenia was refractory to steroids, tacrolimus, mycophenolate mofetil (MMF), and rituximab. In view of the refractory autoimmunity, at 30 months of age, he underwent a matched sibling donor HSCT using fludarabine, treosulfan, and thiotepa (FTT) conditioning, with ciclosporin and methotrexate for graft-versus-host disease (GvHD) prophylaxis. He achieved engraftment at 22 days with donor cell chimerism of 90%. However, there was a rapid decline in donor chimerism to less than 10% within a month, with immune rejection of the graft, autologous haematopoietic reconstitution, and recurrence of primary disease manifesting as monocytosis, splenomegaly, and increasingly severe proteinuria, and resurgent anti-dsDNA antibodies. Renal biopsy confirmed lupus nephritis with extensive glomerular loss, which proved difficult to control with steroids and other immunosuppressants.

A second HSCT was performed from a different sibling donor using fludarabine and busulfan (Flu/BU) conditioning, alemtuzumab serotherapy, and rituximab and ciclosporin for GvHD prophylaxis. He achieved engraftment at 15 days with 100% donor cell chimerism. However, 6 months later, he developed mixed T-cell chimerism of 20%, whilst myeloid chimerism remained fully donor. His autoimmunity recurred, driven by residual recipient T cells carrying the same *NRAS* mutation at diagnosis, as evidenced by chronic immune-mediated thrombocytopenia, lupus nephritis, and rising titres of anti-dsDNA antibodies. His disease did not respond to rituximab, bortezomib, daratumumab, sirolimus, or MMF. Five years after presentation, he was treated with the MEK inhibitor trametinib at a dose of 0.025 mg/kg once daily to specifically target the downstream RAS–MEK–ERK signalling pathway. He showed a significant response, with normalisation of anti-dsDNA antibodies and resolution of proteinuria. Anti-dsDNA titres started rising after 3 months, followed by reappearance of proteinuria and development of an extensive eczematous-type rash, leading to discontinuation of the MEK inhibitor after 6 months. At the time of publication, he remains on a small dose of steroids, MMF, and tacrolimus, with proteinuria limited.

### Patient 2

An 18-month-old man of Asian ethnicity presented with foreign body aspiration and was incidentally found to have DAT-positive autoimmune haemolytic anaemia and severe immune thrombocytopenia. His presenting full blood count demonstrated a haemoglobin of 78 g/L, platelet count of 3 × 10^9^/L, and a total white cell count of 9.7 × 10^9^/L, with monocytosis of 2.4 × 10^9^/L. He had gross hepatosplenomegaly but no lymphadenopathy. Blood film showed leucoerythroblastosis with circulating myeloid and erythroid precursors and monocytosis. HbF was raised with age. Bone marrow assessment revealed a hypercellular marrow with expanded myelopoiesis and no excess blasts. Karyotype was 46,XY, and FISH was negative for BCR-ABL translocation. Myeloid NGS performed on peripheral blood confirmed a heterozygous somatic gain-of-function *KRAS* mutation (p.G13C), which was absent in skin fibroblasts, thereby excluding a germline RAS mutation. No double-negative (CD4/CD8) T-cell population was detected in the T and B lymphocyte subsets in peripheral blood.

Immune cytopenia was managed with steroids, MMF, and rituximab. The clinical course was complicated by an episode of idiopathic pneumonia requiring invasive ventilation. Owing to resistant autoimmune cytopenia, he underwent a mismatched six of eight unrelated cord transplant at 30 months of age, using fludarabine, busulfan, antithymoglobulin (ATG), and rituximab conditioning, with ciclosporin and methylprednisolone as GvHD prophylaxis. He achieved engraftment at 16 days with 99% donor chimerism.

Significant Cytomegalovirus (CMV) reactivation was treated with ganciclovir, followed by rising recipient T cell and myeloid chimerism, with cytopenia and immune-type graft failure 2 months after transplant. He subsequently showed evidence of recurrent disease, with increasing hepatosplenomegaly and a widespread macular rash. At 32 months, he underwent a second transplant with a mismatched six of eight unrelated cord, using fludarabine, reduced-dose treosulfan, and ATG conditioning, with rituximab prophylaxis at D30 and ciclosporin and methylprednisolone prophylaxis against GvHD. He achieved engraftment at 22 days with 100% donor chimerism and remains stable. The posttransplant course was complicated by CMV reactivation, treated with ganciclovir and foscarnet, and colitis resulting in intestinal failure requiring long-term parenteral nutrition. However, 5 years posttransplant, the child is well, with full donor chimerism, free from RALD, and without other comorbidities.

## Discussion

RALD is a rare immune dysregulation syndrome with very few reported cases (1, 3). Autoimmune features, particularly autoimmune cytopenia, are the most common presenting manifestations. Given its rarity and overlapping clinical and laboratory findings with JMML and other PIRD, such as ALPS, RALD frequently represents a diagnostic challenge. **Table 2** outlines the major differential approach. For in-depth analysis of the differential diagnosis, readers are referred to relevant comprehensive reviews (2, 4, 5). Somatic *NRAS* and *KRAS* mutations are identified in a subset of patients with JMML and overlap with mutations described in RALD (2). However, despite this shared molecular aetiology, JMML and RALD are clinically distinct entities. In RALD, *in vitro* T-cell studies have shown partial resistance to interleukin (IL)-2 withdrawal-induced apoptosis with reduced BCL-2-Interacting Mediator of Cell Death (BIM) expression (2), whereas comparable T-cell apoptosis data have not been established as a defining feature of JMML. Importantly, JMML may also be associated with autoimmunity, including hypergammaglobulinaemia and Coombs autoantibodies (6); however, its clinical presentation is primarily that of a myeloproliferative/myelodysplastic neoplasm. Thus, a diagnosis of RALD should be considered when autoimmune features present alongside myeloproliferative manifestations. The two patients in our cohort matched the general profile of RALD described in the literature. Both presented at 18 months of age with autoimmune haemolytic anaemia and immune thrombocytopenia. They also exhibited myeloproliferative features suggestive of JMML, including monocytosis, leucoerythroblastic blood film with circulating myeloid and erythroid precursors, splenomegaly, raised HbF, and hypercellular bone marrow with no excess blasts at presentation. Refractory autoimmunity despite steroids and multiple immunosuppressants was a common theme in both patients, which ultimately became the indication for HSCT.

**Table 2:** Differential diagnostic features of RALD, JMML, ALPS, and juvenile systemic lupus erythematosus (jSLE).

Features	RALD	JMML	ALPS	Juvenile SLE
Underlying mechanism	Somatic gain-of-function *KRAS* or *NRAS*; intrinsic apoptosis/RAS–MAPK dysregulation	Clonal myeloproliferative/myelodysplastic neoplasm driven by RAS-pathway lesions	Defective FAS-mediated extrinsic apoptosis; typically the FAS/FASLG/CASP10 pathway	Systemic autoimmune disease with immune complex formation
Age at onset	Infancy/early childhood	Early childhood	Early childhood	Adolescence
Sex	No difference reported	Slight male predominance	No difference reported	Strong female predominance
Splenomegaly	Common	Common	Common	Occasional
Hepatomegaly	Common	Common	Common	Occasional
Lymphadenopathy	Common	Common	Common	Common, generalised
Skin manifestations	Variable; rash reported, lupus-like features possible	Nonspecific rash occasionally	Usually limited; may have eczema/autoimmune skin disease depending on genotype	Common: malar rash, photosensitivity, vasculitic lesions, discoid lesions
Autoimmune cytopenias	Very common; often presenting feature	Can occur, but are not a dominant feature	Very common	Common
Monocytosis	Very common	Very common; diagnostic feature	Absent	Absent
Neutrophilia	May occur with myeloproliferative phenotype	Common	Absent	Absent (leukopenia is more common)
Renal involvement	Occasional lupus nephritis-like disease	Uncommon	Rare/uncommon	Common; lupus nephritis major morbidity feature
Complement (C3/C4) levels	Commonly normal; may be reduced in autoimmune/lupus-like phenotype	Normal	Normal	Commonly reduced in active disease
ANA	Occasionally positive	Occasionally positive	Usually negative or low titre; may occur in overlap phenotypes	Strongly positive
Anti-dsDNA antibodies	Commonly negative; may be positive in lupus-like RALD	Negative	Negative	Characteristically positive, especially with nephritis
Other biomarkers	CD10+ polytypic B cells; aberrant CD14 expression on granulocytes; IL-2 withdrawal apoptosis decreased in research testing	GM-CSF hypersensitivity	Raised αβ double-negative T cells, soluble FASL, IL-10, vitamin B12; defective FAS-mediated apoptosis	NA
Genetics	Somatic KRAS or NRAS only	PTPN11, KRAS, NRAS, NF1, CBL and others	FAS/FASLG/CASP10 pathway defects most common	Polygenic; monogenic lupus is possible in early-onset disease
Malignancy risk	Possible evolution to JMML or other malignancy	Malignant disease	Increased risk of lymphoma	Uncommon
Response to lupus therapy	Often incomplete/refractory	Not responsive	Cytopenias may respond, but lymphoproliferation persists	Typically, a responsive, refractory disease can occur
Helpful discriminator	Autoimmunity + monocytosis + splenomegaly with somatic KRAS/NRAS and no JMML-defining evolution	Progressive clonal myeloid disease, blasts/dysplasia, monosomy 7 or additional clonal hits	Marked lymphadenopathy/splenomegaly with high DNT and ALPS biomarkers, without monocytosis-driven myeloid picture	Lupus classification features dominate; monocytosis/RAS mutation should prompt RALD/JMML review

*NA*, not available.

**Figure 1:**
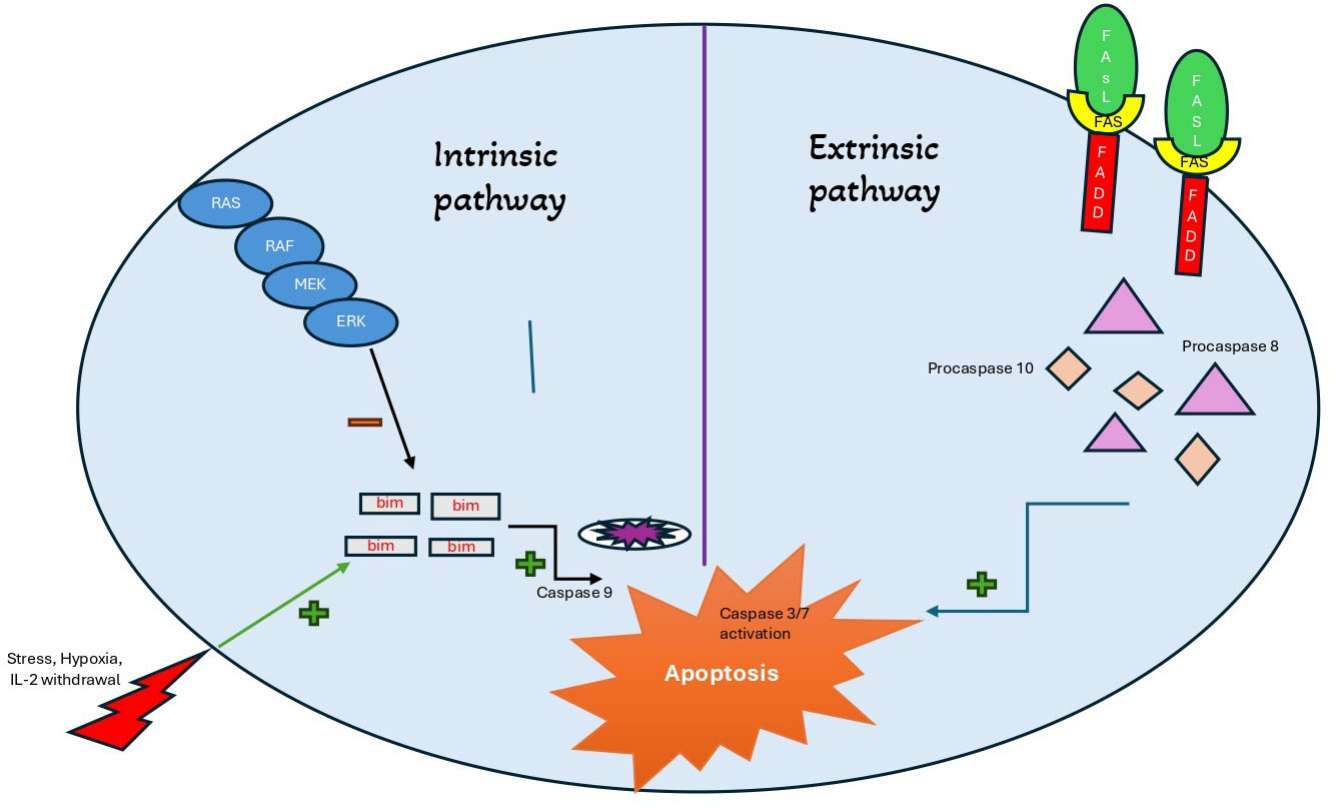
Illustration of two different pathways of apoptosis. Triggers such as hypoxia, DNA damage, and growth cytokine withdrawal activate the intrinsic pathway, leading to increased translocation of the proapoptotic protein *bim* into the mitochondria. This process reduces antiapoptotic proteins and diminishes mitochondrial oxidative phosphorylation. Ultimately, it results in caspase-mediated programmed cell death. In contrast, activation of the RAS–RAF–MEK–ERK signalling pathway reduces *bim* concentration and confers a survival advantage to the cell. The extrinsic pathway is activated through interaction and recruitment of the FAS ligand–FAS–FADD complex, which initiates activation of procaspase 8 and 10, ultimately resulting in cell apoptosis.

Lymphocyte apoptosis is indispensable for maintaining immune homeostasis and tolerance, ensuring a tightly regulated functional lymphocyte repertoire and deletion of self-reactive lymphocytes; without this, autoimmunity would inevitably result (7). Disorders of the extrinsic apoptotic pathway lead to ALPS, whereas disorders of the intrinsic pathway cause RALD (**Figure 1**). RALD, previously designated ALPS type IV (8), is driven by somatic gain-of-function *NRAS* or *KRAS* variants that disrupt interaction with GTPases, activating the downstream RAS–MEK–ERK signalling pathway and leading to phosphorylation and depletion of the proapoptotic protein BIM (9). This renders cells impervious to apoptotic stimuli such as IL-2 withdrawal and triggers autoimmune complications (10). RAS signalling is also involved in B-cell selection, and overactive signalling results in antibody-mediated autoimmunity (11). The FS-7-associated surface antigen (FAS)-mediated apoptotic pathway, which is defective in ALPS, remains intact in this condition.

Trametinib is an orally bioavailable allosteric MEK1/2 inhibitor that has been used effectively in various malignancies driven by abnormal RAS–MAPK signalling, including advanced melanoma, paediatric brain tumours, and other solid tumours (12–14). Following encouraging phenotypic responses in preclinical studies with *NF1-* and *KRAS-*mutated mouse models, the Children’s Oncology Group evaluated trametinib in a phase 2 clinical trial enrolling 10 patients with relapsed and/or refractory advanced JMML, which demonstrated the safety and effectiveness of the drug, with at least 50% objective response in this otherwise dismal condition (15). By analogy, we speculate that MEK inhibitors may have a role in this rare disease, particularly when conventional immunosuppression proves ineffective, either as stand-alone therapy or as a bridge to HSCT. There has been one documented use of a MEK inhibitor in the RALD setting. A 36-year-old patient with *KRAS-*mutated RALD and Rosai–Dorfman disease (RDD) who progressed to chronic myelomonocytic leukaemia (CMML) was successfully treated with cobimetinib to control the disease, enabling safe matched unrelated donor HSCT (16). In our patient, there was a good disease response to trametinib, with the disappearance of anti-dsDNA antibodies and proteinuria for a 3-month period. However, breakthrough relapse occurred on this drug, coupled with excessive grade 3 skin toxicity in the form of a photosensitive erythematous macular rash, which limited its use.

This is amongst the first reported cases of RALD treated with HSCT. They indicate an increased risk of graft rejection, even following myeloablative conditioning and serotherapy. This likely reflects immune activation associated with residual recipient T cells. These residual T cells also sustain disease manifestations, including cytopenia and lupus nephritis in our second case. While mixed T-cell chimerism in the presence of full myeloid chimerism is generally not considered significant in nonmalignant transplants (17), there are a handful of disorders in which it can be problematic, particularly where a gain-of-function mutation occurs in the T-cell lineage, such as in Signal transducer and activator of transcription 1 (STAT1) or Signal transducer and activator of transcription 3 (STAT3) disease.

The two reported HSCT for RALD suffered relatively early graft rejection, and it is tempting to speculate that the less well-researched, inherently nonconducive inflammatory marrow microenvironment may be implicated. Evidence indicates that an inflammatory bone marrow milieu creates an unfavourable environment for engrafting donor stem cells, thereby enhancing graft rejection in many PIRD. In PIRD, including primary HLH, CTLA4 haploinsufficiency, and STAT1 and STAT3 gain-of-function variants, the use of pretransplant biologic modifiers and targeted therapy has been shown to attenuate niche inflammation, mitigate the risk of graft rejection, and improve HSCT survival (18). In a related disorder within the extended spectrum, such as JMML, pretransplant azacitidine has been documented to improve transplant outcomes (19), partly by reducing tumour burden and partly by attenuating associated inflammation. Comparably, addressing and reducing the inflammation linked to autoimmunity in RALD with MEK inhibitors, with or without pretransplant immunosuppression, may improve graft survival in this orphan condition.

We conducted a literature search to identify the haemopoietic stem cell transplants reported in this rare disorder and to examine in detail the indications, pretransplant preparatory regimes (if any), conditioning regimes, graft sources, complications, and the outcomes.

There have been three previous HSCT for malignant transformation in RALD (**Table 3**).

**Table 3:** Demographics and transplant characteristics of RALD patients to date.

Characteristics	Patient 1	Patient 2	Patient 3	Patient 4	Patient 5
Age at presentation	15 months	5 months	Childhood	18 months	18 months
Sex	Female	Female	Female	Male	Male
Type of RAS variant	KRAS p.G13A	KRAS p.G13D	KRAS p.G13C	NRAS p.G13A	KRAS p.G13C
Presentation	Autoimmune cytopenia, monocytosis	Autoimmune cytopenia, splenomegaly	Autoimmune cytopenia, splenomegaly, Rosai–Dorfman disease	Autoimmune cytopenia, splenomegaly, monocytosis	Autoimmune cytopenia, splenomegaly, monocytosis
Indication for transplant	AML transformation from JMML	Refractory autoimmune cytopenia	CMML transformation	Refractory autoimmune cytopenia	Refractory autoimmune cytopenia
Age at the time of transplant	5 years	Unk	36 years	22 months	24 months
Pretransplant therapeutic regimen	AML-directed intensive chemotherapy	Prednisolone, IVIG, low-dose cytarabine	Cobimetinib	Steroids, tacrolimus, MMF, rituximab	Steroids, MMF, rituximab
Conditioning	Flu/Bu/TT	Unk	Unk	FTT	Flu/Bu (MAC)
Donor source	Haploidentical	Unk	MUD	MSD	CB
Posttransplant GvHD prophylaxis	PTCy, tacrolimus, MMF	Unk	Unk	Ciclosporin, methotrexate	Ciclosporin, MMF
Outcome	• Full donor chimerism • Grade 2 GvHD	Unk	Unk	Early graft rejection with relapse of lupus nephritis	Early graft rejection
Details of the second HSCT	n/a	n/a	n/a	Flu/Bu (MAC); MSD	FTT; CB
Outcome after second HSCT	n/a	n/a	n/a	Mixed T-cell chimerism with relapse of lupus nephritis	Full donor chimerism in all lineages with disease remission

*CB*, cord blood; *Flu/Bu/TT*, fludarabine/busulfan/thiotepa; *FTT*, fludarabine/thiotepa/treosulfan; *GvHD*, graft-versus-host disease; *MAC*, myeloablative conditioning; *MMF*, mycophenolate mofetil; *MSD*, matched sibling donor; *MUD*, matched unrelated donor; *PTCy*, posttransplant cyclophosphamide; *Unk*, unknown.

One HSCT was performed in a 5-year-old child who progressed from JMML to Acute Myeloid Leukaemia (AML), after a 4-year follow-up from the diagnosis of RALD (10). Following AML-directed intensive chemotherapy, she underwent HSCT with fludarabine, busulfan, and thiotepa conditioning, and posttransplant cyclophosphamide, tacrolimus, and MMF as GvHD prophylaxis. She developed grade 2 GvHD. The transplant was successful, achieving full donor chimerism without evidence of disease relapse during a 26-month follow-up period.

The other HSCT was undertaken in an infant who presented with Evans’ syndrome, positive ANA and anti-dsDNA, and features of JMML. Owing to incomplete remission of autoimmunity despite treatment with prednisolone, intravenous immunoglobulin, and low-dose cytarabine, stem cell transplant was performed (20). However, the details of the transplant and its outcome were not documented.

The third HSCT was performed in a 36-year-old patient with *KRAS*-mutated RALD with Rosai–Dorfman disease who progressed to CMML, in whom a MEK inhibitor was used as a bridge to a matched unrelated donor HSCT (16). The details of the conditioning and outcome are not clearly documented.

We suggest that HSCT be considered in cases of difficult-to-treat autoimmunity and malignant transformation in RALD. We propose a preparatory regimen comprising a MEK inhibitor with immunosuppression, busulfan-based myeloablative conditioning, and concurrent intensive immunosuppression to achieve full donor chimerism across all lineages, particularly when transplant is done for autoimmunity and in chemotherapy-naive patients.

Further data on HSCT in RALD are required to substantiate the distinctive risks and complications, and to enable measures that mitigate these in transplant settings.

## Conclusion

RALD, although deemed as a chronic nonmalignant condition, has a close aetiologic and clinical relationship with JMML and combines features of malignant haematologic disease and autoimmune manifestations, often refractory to conventional immune suppression. HSCT should, therefore, be regarded as a treatment option. Conditioning in this setting should be both myeloablative and intensely immunosuppressive, and MEK inhibitors may be recommended in refractory cases of RALD before proceeding to stem cell transplant. Our experience provides insight into transplant in this condition, particularly the significance of posttransplant residual recipient T-cell chimerism in sustaining disease manifestations and mediating graft rejection.

## Data Availability

The original contributions presented in the study are included in the article/supplementary material. Further inquiries can be directed to the corresponding author.

## References

[1] Neven Q Boulanger C Bruwier A de Ville de Goyet M Meyts I Moens L . Clinical spectrum of Ras-associated autoimmune leukoproliferative disorder (RALD). J ClinImmunol. (2021) 41:51–8. doi: 10.1007/s10875-020-00883-7 33011939

[2] Calvo KR Price S Braylan RC Oliveira JB Lenardo M Fleisher TA . JMML and RALD (Ras-associated autoimmune leukoproliferative disorder): common genetic etiology yet clinically distinct entities. Blood. (2015) 125:2753–8. doi: 10.1182/blood-2014-11-567917 25691160 PMC4424627

[3] Ren J Wan Y Lan Y Li X Li J Zhang L . Ras-associated autoimmune lymphoproliferative disorder: retrospective case series. Blood. (2024) 144, 1162. doi: 10.1182/blood-2024-206929

[4] Sullivan KE Lambert M . Ras‐associated autoimmune lymphoproliferative disorder. Br J Haematol. (2024) 205:819–22. doi: 10.1111/bjh.19564 38797558

[5] López-Nevado M González-Granado LI Ruiz-García R Pleguezuelo D Cabrera-Marante O Salmón N . Primary immune regulatory disorders with an autoimmune lymphoproliferative syndrome-like phenotype: immunologic evaluation, early diagnosis and management. Front Immunol. (2021) 12. doi: 10.3389/fimmu.2021.671755 34447369 PMC8382720

[6] Wintering A Dvorak CC Stieglitz E Loh ML . Juvenile myelomonocytic leukemia in the molecular era: a clinician’s guide to diagnosis, risk stratification, and treatment. Blood Adv. (2021) 5:4783–93. doi: 10.1182/bloodadvances.2021005117 34525182 PMC8759142

[7] Rathmell JC Thompson CB . Pathways of apoptosis in lymphocyte development, homeostasis, and disease. Cell. (2002) 109:S97–107. doi: 10.1016/s0092-8674(02)00704-3 11983156

[8] Oliveira JB Bidère N Niemela JE Zheng L Sakai K Nix CP . NRAS mutation causes a human autoimmune lymphoproliferative syndrome. Proc Natl Acad Sci USA. (2007) 104:8953–8. doi: 10.1073/pnas.0702975104 17517660 PMC1885609

[9] Ley R Ewings KE Hadfield K Cook SJ . Regulatory phosphorylation of Bim: sorting out the ERK from the JNK. Cell Death Differ. (2005) 12:1008–14. doi: 10.1038/sj.cdd.4401688 15947788

[10] Niemela JE Lu L Fleisher TA Davis J Caminha I Natter M . Somatic KRAS mutations associated with a human nonmalignant syndrome of autoimmunity and abnormal leukocyte homeostasis. Blood. (2011) 117:2883–6. doi: 10.1182/blood-2010-07-295501 21079152 PMC3062298

[11] Teodorovic LS Babolin C Rowland SL Greaves SA Baldwin DP Torres RM . Activation of Ras overcomes B-cell tolerance to promote differentiation of autoreactive B cells and production of autoantibodies. Proc Natl Acad Sci USA. (2014) 111:E2797–806. doi: 10.1073/pnas.1402159111 24958853 PMC4103347

[12] Long GV Stroyakovskiy D Gogas H Levchenko E de Braud F Larkin J . Combined BRAF and MEK inhibition versus BRAF inhibition alone in melanoma. N Engl J Med. (2014) 371:1877–88. doi: 10.1056/NEJMoa1406037 25265492

[13] Bouffet E Hansford JR Garrè ML Hara J Plant-Fox A Aerts I . Dabrafenib plus Trametinib in pediatric glioma with BRAF V600 mutations. Journal of Clinical Oncology. N Engl J Med. (2023) 389:1108–20. doi: 10.1056/NEJMoa2303815 37733309

[14] Gouda MA Subbiah V . Expanding the benefit: Dabrafenib/Trametinib as tissue-agnostic therapy for BRAF V600E-positive adult and pediatric solid tumors. Am Soc Clin Oncol Educ Book. (2023) 43:e404770. doi: 10.1200/EDBK_404770 37159870

[15] Stieglitz E Lee AG Angus SP Davis C Barkauskas DA Hall D . Efficacy of the allosteric MEK inhibitor Trametinib in relapsed and refractory juvenile myelomonocytic leukemia: a report from the Children's Oncology Group. Cancer Discov. (2024) 14:1590–8. doi: 10.1158/2159-8290.CD-23-1376 38867349 PMC11374478

[16] Wilson NR Fang H Loghavi S Wang W Tang G Haltom RO . Treating Rosai-Dorfman disease and RAS-associated autoimmune leucoproliferative disorder with Malignant transformation. Br J Haematol. (2021) 192:667–71. doi: 10.1111/bjh.17258 33238033

[17] Zimmerman C Shenoy S . Chimerism in the realm of hematopoietic stem cell transplantation for non-malignant disorders - a perspective. Front Immunol. (2020) 11:1791. doi: 10.3389/fimmu.2020.01791 32903736 PMC7438804

[18] Arnold DE Chellapandian D Leiding JW . The use of biologic modifiers as a bridge to hematopoietic cell transplantation in primary immune regulatory disorders. Front Immunol. (2021) 12:692219. doi: 10.3389/fimmu.2021.692219 34248986 PMC8264452

[19] Niemeyer CM Flotho C Lipka DB Starý J Rössig C Baruchel A . Response to upfront azacitidine in juvenile myelomonocytic leukemia in the AZA-JMML-001 trial. Blood Adv. (2021) 5:2901–8. doi: 10.1182/bloodadvances.2020004144 34297046 PMC8341358

[20] Takagi M Shinoda K Piao J Mitsuiki N Takagi M Matsuda K . Autoimmune lymphoproliferative syndrome-like disease with somatic KRAS mutation. Blood. (2011) 117:2887–90. doi: 10.1182/blood-2010-08-301515 21063026

